# Epigenetic Modification of Mesenchymal Stromal Cells Derived from Bone Marrow and Embryonal Tumors to Facilitate Immunotherapeutic Approaches in Pediatric Malignancies

**DOI:** 10.3390/cimb45030136

**Published:** 2023-03-03

**Authors:** Anne Kruchen, Pascal-David Johann, Laura Rekowski, Ingo Müller

**Affiliations:** 1 Division of Pediatric Stem Cell Transplantation and Immunology, Clinic of Pediatric Hematology and Oncology, University Medical Center Hamburg-Eppendorf, Martinistr. 52, 20246 Hamburg, Germany; 2 Swabian Children’s Cancer Center, Children’s Hospital, Klinikum Augsburg, Stenglinstr. 2, 86156 Augsburg, Germany; 3 Division of Pediatric Neurooncology, German Cancer Research Center (DKFZ), 69120 Heidelberg, Germany; 4 Hopp Children’s Cancer Center (KiTZ), 69120 Heidelberg, Germany; 5 Department of Pediatric Hematology and Oncology, University Children’s Hospital Heidelberg, 69120 Heidelberg, Germany; 6 German Cancer Consortium (DKTK), German Cancer Research Center (DKFZ), 69120 Heidelberg, Germany; 7 Research Institute Children’s Cancer Center Hamburg, Martinistr. 52, 20251 Hamburg, Germany

**Keywords:** mesenchymal stromal cells, leukemic stem cell niche, tumor microenvironment, histone deacetylase inhibitor

## Abstract

Mesenchymal stromal cells (MSC) are part of the bone marrow architecture and contribute to the homeostasis of hematopoietic stem cells. Moreover, they are known to regulate immune effector cells. These properties of MSC are pivotal under physiologic conditions, and they may aberrantly also protect malignant cells. MSCs are also found in the leukemic stem cell niche of the bone marrow and as part of the tumor microenvironment. Here, they protect malignant cells from chemotherapeutic drugs and from immune effector cells in immunotherapeutic approaches. Modulation of these mechanisms may improve the efficacy of therapeutic regimens. We investigated the effect of the histone deacetylase inhibitor (HDACi) suberoylanilide hydroxamic acid (SAHA, Vorinostat™) on the immunomodulatory effect and cytokine profile of MSC derived from bone marrow and pediatric tumors. The immune phenotype of MSC was not markedly affected. SAHA-treated MSC showed reduced immunomodulatory effects on T cell proliferation and NK cell cytotoxicity. This effect was accompanied by an altered cytokine profile of MSC. While untreated MSC inhibited the production of certain pro-inflammatory cytokines, SAHA treatment led to a partial increase in IFNγ and TNFα secretion. These alterations of the immunosuppressive milieu might be beneficial for immunotherapeutic approaches.

## 1. Introduction

The leukemic stem cell niche in the bone marrow, and likewise the tumor microenvironment, constitute a sanctuary for malignant cells protecting them from attacks by drugs in chemotherapeutic regimens and from immune effector cells in immunotherapeutic approaches. This immune-privileged milieu is established by cross-talk between malignant cells and their surrounding tissue [1].

In healthy individuals, the bone marrow hosts hematopoietic progenitor cells (HPC), which are home to and engraft in highly specific niches regulating their survival, proliferation, and differentiation [2,3]. Bone marrow-derived mesenchymal stromal cells (BM-MSC) exert many-facetted immunosuppressive effects, modulating functions of B cells and dendritic cells, and in particular, inhibiting immune effector cell proliferation and cytotoxic activity [4,5,6,7]. By inhibiting the production of tumor necrosis factor alpha (TNFα) and interferon-gamma (IFNγ), while increasing the production of interleukin-10 (IL-10), BM-MSC promotes T helper 2 (T_H_2) cell responses. The clinical use of BM-MSC ranges from tissue engineering to immunotherapies [8,9]. MSC is widely used in Europe and Japan for the treatment of graft versus host disease (GvHD) and Crohn’s fistular disease [10].

In hematologic malignancies, leukemic cells enter the bone marrow and create leukemic stem cell niches that disrupt the behavior of normal HPC [2]. The bone marrow stromal microenvironment, e.g., contributes to the survival of chronic lymphocytic leukemia (CLL) cells, leading to drug resistance in vivo [1,11]. In mouse models, it was shown that acute myeloid leukemia (AML) blasts utilized exosome secretion to remodel the bone marrow niche. AML-derived exosomes increased mesenchymal stromal progenitors and blocked osteolineage development as well as bone formation [12]. BM-MSC from patients with myelodysplastic syndrome (MDS) showed chromosomal abnormalities but retained their potential to differentiate. Production of IL-1β, IL-6, and TNF-α was increased [13,14]. In vitro co-cultures of BM-MSC and patient-derived MDS cells triggered the overproduction of certain niche factors that are associated with the ability of MDS-MSC to enhance MDS expansion [15].

Similar to leukemic blasts in the niche, solid tumor cells are embedded in a microenvironment of tumor endothelial cells, tumor-associated macrophages, and tumor-derived mesenchymal stromal cells (T-MSC). Like BM-MSC, T-MSC compromises the immune effector functions against malignant cells [16]. T-MSC resembles BM-MSC in morphological, phenotypical, and functional aspects [17]. Hence, BM-MSC is considered to be the source of T-MSC, and the homing of BM-MSC in tumor sites has been extensively studied [18,19,20]. Therapeutic strategies can target both the malignant cells and their protective environment, thereby possibly circumventing the MSC-mediated protection.

Since epigenetic alterations and mutations in epigenetic regulators are known to contribute to the pathogenesis of AML, the use of epigenetic inhibitors such as histone deacetylase inhibitors (HDACi) is under clinical development [21]. Suberoylanilide hydroxamic acid (SAHA, Vorinostat™, Zolinza^®^), a pan HDACi, was approved by the FDA (United States Food and Drug Administration) for the treatment of cutaneous T-cell lymphoma (CTCL) in 2006 [22]. By binding to the active site of HDAC, SAHA impacts several genes and proteins promoting cell cycle arrest, apoptosis, and differentiation, especially of transformed cells. Preclinical studies have demonstrated the potential antitumor activity of SAHA against leukemic cells and other hematologic malignancies in vitro, and improved survival in animal models of leukemia [23,24]. Mechanistically, upregulation of pro-apoptotic genes, e.g., p53, and downregulation of certain anti-apoptotic genes, e.g., Bcl-2, have been described [25,26]. Intriguingly, the administered concentration impacts the exerted effect of SAHA. For antitumor effects, micromolar concentrations are required, whereas at nanomolar concentrations the secretions of pro-inflammatory cytokines, such as TNFα, IFNγ, IL-1β, and IL-12 by LPS-induced peripheral blood mononuclear cells (PBMC) are decreased [27].

Nanomolar concentrations of SAHA were also sufficient to enhance the efficacy of chimeric antigen receptor (CAR) T cells against solid tumors including downregulating the expression of immunosuppressive molecules such as CTLA-4 [28]. Furthermore, tumor-invading T cells as well as CD8^+^ T cells in the bone marrow of AML patients exhibit an exhausted phenotype, e.g., an increased expression of PD1. T cell escape mechanisms by malignant cells include the downregulation of major histocompatibility class I (MHC-I) molecules. Neuroblastoma and other tumors arising from embryonic tissues show an intrinsic lack of MHC-I expression resulting from epigenetic and/or (post)transcriptional processes, which can be reversible [29]. Treatment of neuroblastoma cells with the HDACi entinostat was effective to upregulate or restore MHC-I expression, and tumor-specific T cells showed an increased cytotoxic activity [30]. A phase I/II clinical study combining entinostat and the immune checkpoint inhibitor nivolumab in children and adolescents with high-risk refractory malignancies (NCT03838042).

Taken together, these findings propose the question of if HDACi treatment might also beneficially impact immunosuppressive milieus [31,32].

MSC in the leukemic stem cell niche and in the tumor environment is an attractive target for antineoplastic therapy. To further investigate the signaling between the microenvironment and immune effector cells, we analyzed the effect of SAHA on the immunomodulatory properties of MSC derived from bone marrow and pediatric tumors.

## 2. Materials and Methods

### 2.1. Cell Culture and Isolation of Tumor-Derived Mesenchymal Stromal Cells

Tumor tissue from neuroblastoma patients was obtained from residual material after pathological analysis. Informed written consent was obtained from the parents and the protocol was approved by the local IRB (892007V). Histological diagnosis was confirmed by the Institute of Pathology, University of Tübingen. Tumor tissue was disrupted mechanically and placed in 2 mL DMEM medium containing 1 g/L glucose (LG-DMEM, Lonza, Basel, Switzerland), supplemented with 5% (*v/v*) human fresh frozen plasma (FFP), 10^7^/mL platelets (both University of Tübingen blood donor center), 80 IU/mL heparin sulfate (Medunasal, Isemhagen, Germany), 100 IU/mL penicillin and 100 mg/mL streptomycin (Biochrom, Berlin, Germany), 2 mM glutamine (Biochrom, Berlin, Germany) and incubated at 37 °C with 10% CO_2_. T-MSC colonies appeared after 7–9 days. Non-adherent cells were removed, and adherent cells were detached using trypsin (Lonza, Basel, Switzerland) when confluent [17].

Specimens of BM-MSC were derived from excess material of standard bone marrow biopsies in children treated for leukemia (IRB approval 241/2005V). MSC cultures were established as reported earlier [33].

### 2.2. CFSE Proliferation Assays

The immunomodulatory features of T- and BM-MSC on PBMC were analyzed by CFSE proliferation assays. The indicated numbers of MSC were seeded per well in a 96-well plate (Greiner, Frickenhausen, Germany). HLA-mismatched PBMC were obtained from healthy volunteers (IRB approval 892007V). PBMC were stained with CFSE according to the manufacturer’s protocol (Invitrogen, Eugene, OR, USA) and stimulated with 100 U/mL IL-2 (BD Biosciences, Heidelberg, Germany) and 1 μg/mL OKT3 ((Janssen-Cilag, Neuss, Germany), as described previously [33]. PBMCs at 75 × 10^4^ were added per well. Proliferation of PBMC was analyzed by flow cytometry on days five to six.

The influence of SAHA treatment on the immunomodulatory features of MSC was analyzed by CFSE proliferation assays, likewise. MSCs were treated with SAHA for two days prior to co-cultivation with PBMC. A 100 μM working solution of SAHA (Vorinostat^®^, Merck, Darmstadt, Germany) was prepared containing 1% DMSO (Sigma-Aldrich/Merck KGaA, Darmstadt, Germany), and all subsequent dilutions were prepared in cell culture medium. All SAHA concentrations and controls (0 µM) contained the same amount of DMSO in total in each experiment. MSCs were seeded into a 6-well plate (Greiner, Frickenhausen, Germany) and treated with 0.1–5 µM SAHA or corresponding controls for 48 h. Cells were then washed, trypsinized, and counted in a Neubauer chamber. The indicated numbers of MSC were subsequently seeded per well in a 96-well plate (Greiner, Frickenhausen, Germany) and CFSE proliferation assays were performed as described above for non-treated MSC/PBMC co-cultivations.

### 2.3. Cytokine Profiling

For cytokine profiling, the indicated numbers of untreated, control-treated, or SAHA-treated MSC were seeded in 96-well-plates and co-cultivated with IL-2/OKT3-stimulated PBMC as described for CFSE proliferation assays (Section 2.2). After 48 h of co-cultivation, 100 μL supernatant was collected and centrifuged for 10 min at 13,000× *g* at 4 °C. Probes were stored at −20 °C until they were analyzed using the Bio-Plex Pro™ Cytokine profiling assay (Bio-Rad Laboratories, Hercules, CA, USA). The concentrations of IL-1β, IL-2, IL-4, IL-10, IL-12(p70), IL-15, IL-17, INF-γ, and TNF-α were determined according to the manufacturer`s protocol in a Bio-Plex 200 system and evaluated using the integrated software. Detection ranges for each cytokine based on standard curves were: 0.7–2753 pg/mL for IL-1β, 1.27–24,906 pg/mL for IL-2, 1.26–5015 pg/mL for IL-4, 0.56–6912 pg/mL for IL-10, 0.45–39,995 pg/mL for IL12p70, 0.45–31,971 pg/mL for IL-15, 1.31–31,233 pg/mL for IL-17, 24.29–26,485 pg/mL for IFNγ, and 1.88–81,837 pg/mL for TNFα.

### 2.4. Flow Cytometry

Flow cytometric analysis was performed on a FACS Calibur (Becton Dickinson, Heidelberg, Germany) and data were analyzed with CellQuest software (Becton Dickinson, Heidelberg, Germany). Anti-CD73-PE (A02) antibody was purchased from Becton Dickinson, Heidelberg, Germany, anti-CD105-FITC (N1-3A1) from Ancell, Bayport, MN, USA, and anti-CD90-PE (F15-42-1) from Serotec, Düsseldorf, Germany. Isotype controls FITC and PE (both MOPC-21) were purchased from Biolegend, San Diego, CA, USA.

### 2.5. MTS Proliferation Assays

The effect of SAHA on cell proliferation of MSC was examined using the CellTiter 96^®^ Aqueous One Solution Cell Proliferation Assay (Promega, Madison, WI, USA). Prior to the assay 1 × 10^4^, MSCs were seeded in 100 μL per well in a 96-well plate (Greiner, Frickenhausen, Germany). After 2 h cells became adherent and were then treated with 0.1–5 μM SAHA or corresponding controls for 48 h. MTS assays were performed according to the manufacturer`s protocol. Read-out of fluorescence intensity was performed using a Milenia Kinetic Analyzer ELISA Reader (Diagnostic Products Corporation, Software SOFTmax PRO, Molecular Devices Corporation, Sunnyvale, CA, USA).

### 2.6. BATDA Cytotoxicity Assay

The effect of SAHA-treated MSC on NK cell function was assessed by BATDA cytotoxicity assays as described previously [34]. MSCs were seeded into a 24-well plate and treated with 0.1–3.5 μM SAHA or corresponding controls for 48 h. NK cells were isolated by immunomagnetic selection using CD56^+^ magnetic beads (Miltenyi Biotech, Bergisch Gladbach, Germany) from PBMC of healthy donors. Prior to co-cultivation, SAHA-treated MSCs were washed with PBS (Biochrom, Berlin, Germany). NK cells were co-cultured with MSC at a ratio of 4:1 in the presence of 100 U/mL IL-2 (BD Biosciences, Heidelberg, Germany) where indicated. After four days of co-culture, NK cells were isolated and counted in a Neubauer chamber. The leukemic cell line K562 (ATCC) was used as target cell line. K562 was loaded with BATDA according to the manufacturer`s protocol (DELFIA Cell Cytotoxicity kit, PerkinElmer LAS, Rodgau-Jügesheim, Germany). NK cell cytotoxicity against the leukemic cell line K562 (ATCC, Manassas, VA, USA) was tested in triplicates. Effector cells and target cells were incubated for 2 h. Maximum lysis was reached using DELFIA Lysis buffer (PerkinElmer LAS, Rodgau-Jügesheim, Germany). Specific lysis was calculated using the following formula:

$$\%\;\mathrm{Specific}\;\mathrm{lysis}=\frac{\mathrm{Lysis}\;\mathrm{by}\;\mathrm{effector}\;\mathrm{cells}-\mathrm{spontaneous}\;\mathrm{lysis}}{\mathrm{Maximum}\;\mathrm{lysis}-\mathrm{spontaneous}\;\mathrm{lysis}}\times 100$$

### 2.7. Statistical Analysis

Analysis of significance was performed with an unpaired, two-tailed Student’s *t* test or two-way ANOVA with Tukey’s multiple comparisons using GraphPad Prism 9 (San Diego, CA, USA). *p* values of 0.05 or less were considered statistically significant and indicated by asterisks, * *p* < 0.05, ** *p* < 0.01, *** *p* < 0.001, **** *p* < 0.0001.

## 3. Results

### 3.1. T-MSC Show Immunomodulatory Effects on Immune Effector Cells

**Figure 1:**
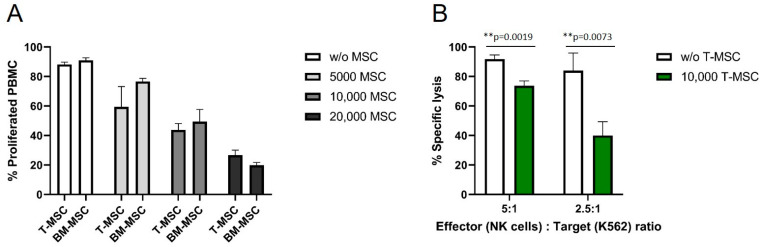
T-MSC reduces the proliferation of IL-2/OKT3-stimulated PBMC comparable to BM-MSC and suppresses the cytotoxicity of NK cells. CFSE proliferation assays of PBMC in the presence or absence of (**A**) T-MSC or BM-MSC. (**B**) NK cells were freshly isolated from peripheral blood, activated with 100 U/mL IL-2 and either cultured alone (white bars) or in the presence of T-MSC (green bars) at a ratio of 4:1 (NK cells: T-MSC) for four days. All results show example experiments with *n* = 3 and SD. ** *p* < 0.01 in an unpaired Student’s *t* test or two-way ANOVA with Tukey’s multiple comparisons. T-MSC: tumor-derived mesenchymal stromal cell; BM-MSC: bone marrow-derived mesenchymal stromal cell.

The antiproliferative effects of T-MSC on effector cells of the immune system were compared to the well-described antiproliferative effects of BM-MSC. HLA-mismatched PBMC were isolated from healthy donors, labeled with CFSE, and co-cultured with MSC for six days. The proliferation of IL-2/OKT3-stimulated PBMC was strongly influenced by T-MSC in a dose-dependent manner (Figure 1A). After six days, more than 90% of stimulated PBMC proliferated in the absence of T-MSC. In co-culture with 5 × 10^3^ T-MSC, 59% of the PBMC proliferated, with 1 × 10^4^ T-MSC 44%, and with 2 × 10^4^ T-MSC 27% of PBMC were still able to proliferate. These inhibitory effects were comparable to the inhibition by BM-MSC (Figure 1A). A two-way ANOVA with Tukey’s multiple comparisons test showed no differences between T-MSC and BM-MSC for each dose, but dose-dependent effects compared to the controls (w/o MSC).

In order to test if the functional properties of immune effector cells are hampered by T-MSC, the cytotoxicity of IL-2-activated primary NK cells against the standard target cell line K562 was analyzed. The specific lysis of NK cells co-cultivated with T-MSC was substantially reduced by more than 50% (E:T 2.5:1), and 20% (E:T 5:1) compared to NK cells without prior co-cultivation (Figure 1B).

### 3.2. T-MSC Alter the Cytokine Profile of PBMC

**Figure 2:**
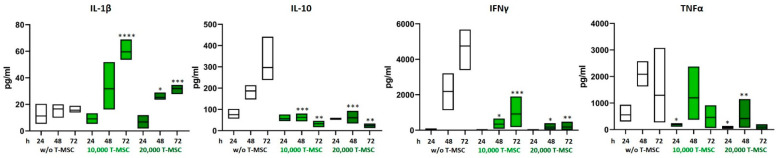
Altered production of pro-inflammatory cytokines after co-cultivation with T-MSC. PBMC were stimulated with IL-2/OKT3 and co-cultivated with T-MSC. Supernatants of co-cultures were analyzed after 24, 48, and 72 h. The cytokine levels were determined by Multiplex analyses. Boxplots show distribution and mean, *n* = 4; * *p* < 0.05, ** *p* < 0.01, *** *p* < 0.001, **** *p* < 0.0001 in an unpaired Student’s *t* test (cytokine value with T-MSC vs. w/o T-MSC for each time point). T-MSC: tumor-derived mesenchymal stromal cell; IL: interleukin; IFNγ: interferon-gamma; TNFα: tumor necrosis factor alpha.

The concentrations of the pro- and anti-inflammatory cytokines IL-1β, IL-2, IL-4, IL-10, IL-12p70, IL-15, IL-17, TNFα, and IFNγ in supernatants from T-MSC-PBMC co-cultivations were determined simultaneously by Multiplex analysis after 24, 48, and 72 h. PBMCs were stimulated with IL-2/OKT3. In a monoculture of T-MSC, the concentrations of cytokines produced stayed below the detection level of the assay (0.1 pg/mL). Hence, the measured data display the PBMC cytokine profiles. The assay was performed with PBMC from two different healthy donors and T-MSC from one source. The concentrations of IL-1β and IL-15 increased from 15.6 to 31.9 pg/mL and from not detectable to 3.2 pg/mL when PBMCs were co-cultivated with T-MSC. Production of IL-10, IL12p70, IFNγ, and TNFα decreased (IL-10 from 297 to 23 pg/mL, IL12p70 from 3.8 pg/mL to not detectable, IFNγ from 4754 to 350 pg/mL, TNFα from 1294 to 128 pg/mL) (Table 1 and Figure 2). IL-17 concentration increased during the first 48 h regardless of co-cultivation with T-MSC. After 72 h, the production of IL-17 stayed stable in co-cultures with 10,000 T-MSC (from 2490 to 3057 pg/mL), was inhibited in co-cultures with 20,000 T-MSC (from 3818 to 1344 pg/mL) but increased without T-MSC (from 1269 to 7684 pg/mL). The secretion of IL-1β was higher in co-cultivation with 1 × 10^4^ T-MSC than with 2 × 10^4^ T-MSC.

### 3.3. Immunophenotype of MSC Is Not Affected by Low Concentrations of SAHA

**Figure 3:**
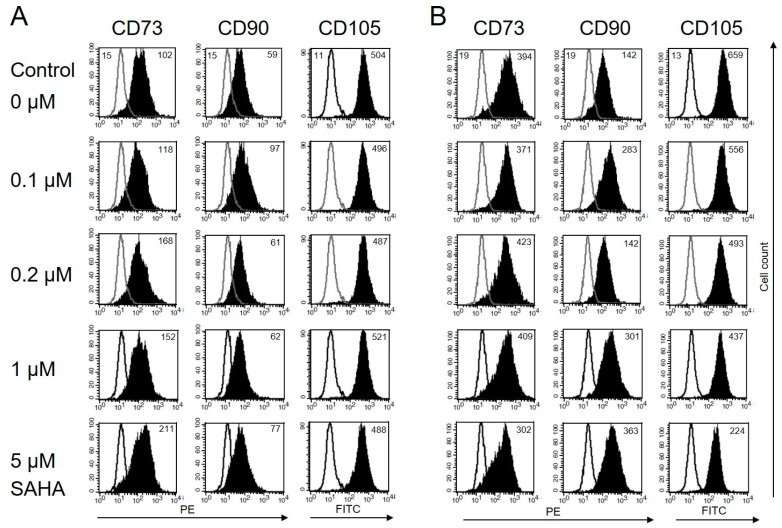
Immune phenotype of MSC is not markedly affected by SAHA treatment. (**A**) Flow cytometric analysis of cell surface markers on T-MSC. T-MSC were treated with SAHA for two days. Isotype controls are depicted in solid lines, and CD73, CD90, and CD105 stainings are depicted in filled histograms. Numbers show the mean fluorescence intensity (MFI). The expression of the surface marker CD73 moderately increased with increasing SAHA concentrations. Expression of CD90 and CD105 was not markedly affected. (**B**) BM-MSC showed an increased expression of CD90, a reduced expression of CD105 with increasing SAHA concentrations, and an unaltered expression of CD73 up to 1 µM SAHA. SAHA: suberoylanilide hydroxamic acid.

Some histone deacetylase inhibitors, e. g. retinoic acid, induce the differentiation of tumor cells. Neuroblastoma cells, for example, show neurite outgrowth. Other HDACi restore MHC-I expression on malignant cells of embryonic origin [29] Such an effect has not been described for MSC, so far. Both, BM-MSC and T-MSC, express CD73, CD90, and CD105 on their surface. The HDACi SAHA is known to exert different effects depending on the administered dose. To assess changes in their morphology and immune phenotype, the cells were treated with nanomolar to micromolar concentrations of SAHA for two days and stained with anti-CD73, anti-CD90, and anti-CD105 antibodies. In the presence of SAHA, BM-MSC and T-MSC retained their fibroblastic shape. The expression of CD90 on T-MSC was not affected by SAHA at concentrations up to 5 μM (Figure 3A). CD73 expression slightly increased with increasing SAHA concentrations. The immune phenotype of BM-MSC was also not significantly altered but showed a slightly increased expression of CD90 and a slightly decreased expression of CD105 under treatment with 5 µM SAHA (Figure 2B).

### 3.4. SAHA Exerts an Antiproliferative Effect on PBMC and MSC

**Figure 4:**
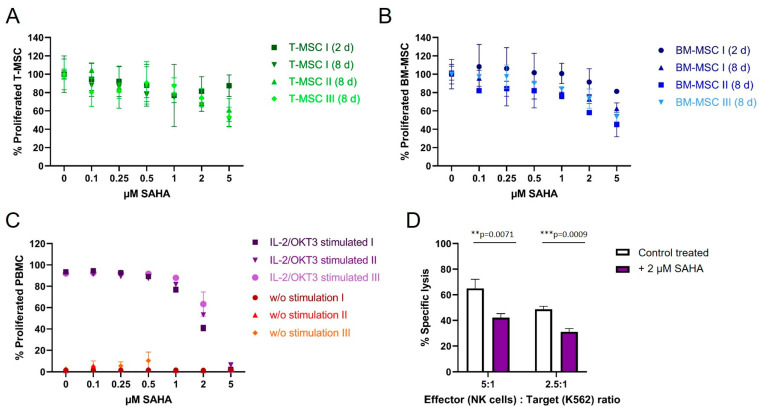
The proliferation of MSC and PBMC was not hampered by nanomolar concentrations of SAHA. (**A**) T-MSCs were treated with SAHA for two or eight days. Proliferation was measured by MTS assays. SAHA concentrations below 1 μM did not severely inhibit the proliferation of T-MSC. (**B**) BM-MSC were treated with SAHA for two or eight days. Proliferation was measured by MTS assays. SAHA concentrations below 0.5 μM did not severely inhibit the proliferation of BM-MSC. (**C**) PBMC were labeled with CFSE, stimulated with IL-2/OKT3, and treated with SAHA for six days. Proliferation was analyzed by flow cytometry. PBMC tolerated SAHA concentrations below 1 μM. (**D**) NK cells were isolated from peripheral blood, activated with 100 U/mL IL-2 overnight, and either cultured alone (control, white bars) or treated with 2 µM SAHA for four days (purple bars). NK cell-specific lysis was analyzed with a BATDA cytotoxicity assay. ** *p* < 0.01, *** *p* < 0.001 in an unpaired Student’s *t* test. PBMC: peripheral blood mononuclear cell, SAHA: suberoylanilide hydroxamic acid; T-MSC: tumor-derived mesenchymal stromal cell; BM-MSC: bone marrow-derived mesenchymal stromal cell; IL: interleukin.

To compare the antiproliferative effect of SAHA on immune effector cells and immunomodulating MSC, cells were treated with the compound between two and eight days. Cell viability was either determined by CFSE (for PBMC) or MTS proliferation assays (T-MSC and BM-MSC). Both T-MSC and BM-MSC (Figure 4A,B, respectively), tolerated higher doses of SAHA compared to PBMC. At a concentration of 5 µM 50–60% of T-MSC and 40–60% of BM-MSC proliferated after 8 days of treatment. At nanomolar concentrations, up to 500 nM, both MSC types were only slightly affected by SAHA treatment and more than 80% of the cells proliferated. Interestingly, T-MSC were equally affected after two days of treatment compared with eight days of treatment at lower SAHA concentrations, while BM-MSC generally better tolerated a shorter treatment time and were more affected after eight days of SAHA treatment.

PBMC from healthy donors were stimulated with IL-2/OKT3 and incubated with SAHA for six days. The proliferation of PBMC was strongly affected compared to MSC (Figure 3C). At a concentration of 2 µM SAHA, only 55% of PBMC still proliferated, higher concentrations resulted in a further reduced proliferation and cell death at 5 µM. Freshly isolated and IL-2-activated NK cells from healthy donors were treated with 2 µM SAHA for four days. SAHA-treated NK cells showed a significantly decreased specific lysis of K562 target cells (Figure 4D). At an E:T ratio of 5:1 NK cells lysed 25% fewer target cells, at an E:T ratio of 2.5:1 NK cells lysed 15% fewer K562 cells compared to untreated cells.

### 3.5. SAHA Treatment Impairs the Immunomodulatory Effects of MSC

**Figure 5:**
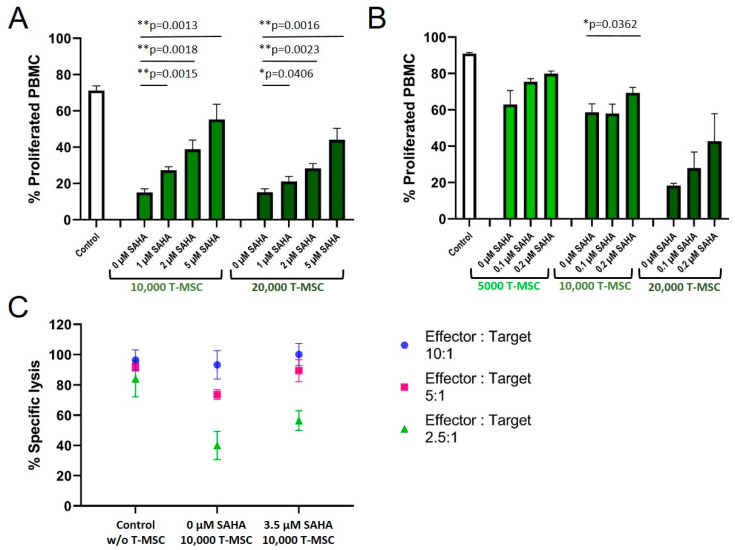
SAHA treatment partially reduced the antiproliferative effects of T-MSC on PBMC. Proliferation of IL-2/OKT3 stimulated PBMC in co-cultures with T-MSC and previously SAHA-treated T-MSC was analyzed by CFSE assays (**A**,**B**). NK cell cytotoxicity in co-cultures with T-MSC and previously SAHA-treated T-MSC was analyzed by BATDA cytotoxicity assays (**C**). NK cells were isolated from peripheral blood, activated with 100 U/mL IL-2 overnight, and either cultured alone (control), in the presence of control-treated T-MSC or T-MSC that had been previously treated with SAHA for four days. Diagrams show mean and SD, *n* = 3. * *p* < 0.05, ** *p* < 0.01 in an unpaired Student’s *t* test. PBMC: peripheral blood mononuclear cell, SAHA: suberoylanilide hydroxamic acid; T-MSC: tumor-derived mesenchymal stromal cell.

A treatment of T-MSC with SAHA for two days resulted in a concentration-dependent partially restored proliferation rate of PBMC in co-cultures (Figure 5A). Control-treated T-MSC decreased the proliferation of PBMC to below 20%. SAHA-treated T-MSC suppressed the proliferation inversely with increasing SAHA treatment (30% at 1 µM, 40% at 2 µM, 55% at 5 µM for 1 × 10^4^ T-MSC; similar for 2 × 10^4^ T-MSC). Since treatment with nanomolar concentrations of SAHA hampered the proliferation of T-MSC only negligibly, concentrations below 0.5 µM were chosen for the next experiment to exclude biasing effects on T-MSC proliferation.

Treatment with 0.1 and 0.2 µM SAHA also compromised the antiproliferative effect of T-MSC on PBMC (Figure 5B). Proliferation rates of PBMC increased from 63 to 80% (5 × 10^3^ T-MSC), from 58 to 69% (1 × 10^4^ T-MSC), and from 18 to 43% (2 × 10^4^ T-MSC). Next, we tested if SAHA treatment also impaired the ability of T-MSC to decrease NK cell cytotoxicity (Figure 5C). T-MSCs were treated with SAHA for two days. SAHA was removed by washing prior to co-cultivation for four days with IL-2-stimulated NK cells. Comparable to the reconstituted proliferation of PBMC, NK cells partially regained their cytotoxic potential against the target cells K562 when co-cultivated with SAHA-treated T-MSC. The specific lysis raised from 75 and 40% (E:T 5:1 and 2.5:1, respectively), after T-MSC-co-culture to 90 and 55% after SAHA-treated T-MSC-co-culture.

### 3.6. SAHA-Treated MSC Alter the Cytokine Profile of PBMC

**Figure 6:**
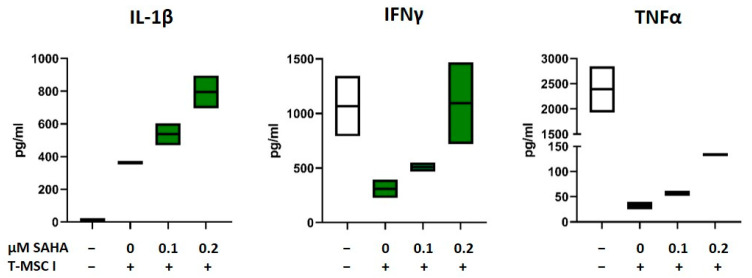
Production of IL-1β, IFNγ, and TNFα after co-cultivation with SAHA-treated T-MSC. PBMC were stimulated with IL-2/OKT3 and co-cultivated without (-), with control-treated T-MSC or previously SAHA-treated T-MSC. Supernatants of co-cultures were analyzed after 48 h. The cytokine levels were determined by Multiplex analyses. PBMCs of two different healthy donors were co-cultivated with T-MSC of two different origins in four separate experiments. Boxplots show distribution and mean of one example experiment for each cytokine, *n* = 2. T-MSC: tumor-derived mesenchymal stromal cell; IL: interleukin; IFNγ: interferon-gamma; TNFα: tumor necrosis factor alpha; SAHA: suberoylanilide hydroxamic acid.

As depicted in Figure 1 and Figure 2 and Table 1, the immunomodulatory capabilities of T-MSC involve an effect on the cytokine production of PBMC. In order to investigate if SAHA treatment has an impact on this alteration, the concentrations of the cytokines IL-1β, IL-2, IL-10, IL-17, TNFα, and IFNγ in supernatants from T-MSC-PBMC co-cultivations were determined simultaneously by Multiplex analysis. PBMCs were stimulated with IL-2/OKT3. T-MSCs were exposed to SAHA for two days prior to co-cultivation. The production of IL-1β by PBMC increased when PBMCs were co-cultivated with T-MSC (Table 1). This increase was even more pronounced when T-MSCs were treated with SAHA (from 335 to 506 pg/mL, Table 2, and exemplary assays depicted in Figure 6). Production of IL-10 and IL-2 was not markedly influenced by SAHA treatment of T-MSC (Table 2). In contrast, the concentrations of TNFα and IFNγ were reduced by co-cultivation. Here, SAHA treatment of T-MSC resulted in a partial restoration of the cytokine production (IFNγ from 124 to 427 pg/mL, and TNFα from 11.5 to 30.5 pg/mL, Table 2 and exemplary assays depicted in Figure 6). The production of IL-17 was divergently influenced. IL-17 levels were decreased under co-cultivation with T-MSC I but increased with T-MSC II.

## 4. Discussion

The persistence of malignant cells strongly depends on the local environment, i.e., altered niches in the bone marrow hosting leukemic stem cells, and tumor stroma surrounding solid tumor cells. In addition, the microenvironment may protect neoplasm from chemotherapy or immunotherapeutic attacks. Consequently, re-remodeling the microenvironment might counteract these protective effects.

We propagated MSC derived from pediatric tumor tissue as well as from the bone marrow of leukemia patients and analyzed their immunomodulatory capacity in the absence or presence of treatment. T-MSC exhibited strong inhibitory effects on immune effector cells. The proliferation of PBMC, as well as NK cell cytotoxicity were profoundly inhibited in co-culture with T-MSC in a cell dose-dependent fashion. These results were in line with previously published data for BM-MSC and T-MSC [4,5,7,17,35]. The production of the pro-inflammatory cytokines IFNγ and TNFα by PBMC was markedly reduced in co-cultivation with T-MSC. Interestingly, IL-10 production was also strongly inhibited by T-MSC. Decreased secretion of IFNγ and TNFα by PBMC in co-culture with MSC was observed uniformly, while secretion of IL-10 was dependent on the pre-activation of PBMC in vitro. αCD28/OKT3-stimulation resulted in increased IL-10 production, whereas IL-2/OKT3 stimulation resulted in decreased IL-10 production [36]. IL-10 is a potent anti-inflammatory cytokine, inhibiting T cell proliferation and the production of pro-inflammatory cytokines. Yet, IL-10 proved not crucial for immunomodulatory effects here.

Various studies demonstrated that MSC derived from patients with leukemia or MDS have altered secretion patterns and surface protein expression phenotypes, that confer survival benefits, immunomodulation, or even chemotherapy resistance to leukemic cells [15,37,38,39,40]. Moreover, Poon et al. showed that BM-MSC from MDS patients exhibit epigenetic abnormalities, suggesting that healthy stromal cells might be epigenetically reprogrammed through exposure to a stimulatory MDS milieu [41]. Reprogrammed stromal cells then might cooperate with leukemic cells propagating the disease all in all.

Hence, therapeutic strategies targeting epigenetic alterations of malignancy-supportive stromal tissues are of high clinical interest. Following treatment with a hypomethylating agent (5-azacitidine, Vidaza^®^), high-risk MDS samples clustered more closely with untreated healthy samples again in a methylation array and lost their ability to impair the growth and function of healthy HPC [41]. We wondered if other epigenetic effects may be exploited in the treatment of pediatric malignancies.

We employed the deacetylating agent SAHA, which did not alter the morphology or immunophenotype of T-MSC. Compared to PBMC, and the effective concentrations reported by others, T-MSC tolerated higher concentrations of SAHA [27]. After SAHA treatment, T-MSC partially lost its antiproliferative impact on PBMC. The exerted effects of SAHA treatment were reported to be concentration dependent, which we also observed [27]. To restore NK cell cytotoxicity partially, micromolar concentrations were required. Moreover, nanomolar concentrations were sufficient to increase PBMC proliferation.

In preclinical studies, SAHA demonstrated anti-tumor activity against leukemia cells by promoting cell death, autophagy, apoptosis, or growth arrest [23,24,42]. Nevertheless, in clinical trials, SAHA displayed restricted anti-tumor activity in CLL patients [1,43]. The drug-resistant survival of CLL cells was attributed to stromal cell-induced antioxidant defense mechanisms [44]. However, higher concentrations of SAHA also resulted in more adverse events and organ toxicities.

Low concentrations of SAHA were reported to exhibit significant anti-inflammatory properties, i.e., reduction of TNFα, IFNγ, and IL1β in a murine GvHD model [45]. In our study, we administered much less, i.e., 100–200 nM, and made different observations in vitro. T-MSC treated with nanomolar concentrations of SAHA led to an altered cytokine profile of co-cultured PBMC as compared to co-cultivation with control-treated T-MSC. Interestingly for immunotherapeutic approaches, the inhibited production of IFNγ and TNFα was partially revoked. Secretion of IL-1β, on the other hand, was further increased after SAHA treatment. These findings suggest that therapeutic approaches are feasible that mainly alter the stromal microenvironment, leaving immune effector cell function unimpaired by the treatment. Since MSC exploits several molecules to interfere with immune effector cell function, e.g., TGF-β, IDO, PGE2, IL-10, HLA-G, Galectin-3, or Galectin-9 [46], specific effects of SAHA on these pathways require further studies.

The interaction of stromal elements and malignant cells has been addressed in different promising approaches, e.g., the detachment of leukemic stem cells from their niche. The chemosensitivity of leukemia cells was increased by the inhibition of E-selectin with its antagonist GMI-1271 [47]. NOS (nitric oxide synthase) inhibitors target the bone marrow vasculature improving chemotherapy efficacy [48]. MSCs exert some functions via the secretion of exosomes. Therefore, targeting exosomes also became an innovative treatment strategy [49].

Systemic treatment with epigenetic modifiers will not only affect stromal elements and malignant cells in the bone marrow but also residing HSC. The expansion of cord blood-derived HSC could be enhanced by treatment with HDACi [50]. In particular, inhibition of HDAC5 evoked an increased surface expression of CXCR4 on HSC leading to enhanced chemotaxis and bone marrow homing [51]. Shim et al. recently demonstrated that the addition of SAHA to embryonic body-derived pluripotent stem cell cultures resulted in higher differentiation rates into HSPCs, accompanied by elevated expression of seven key transcription factors [52]. Our study focused on stromal cells only. We recommend that further studies should include the effect of SAHA treatment on bone marrow-derived HSC regarding the regulation of transcription factors, differentiation potential, as well as the functionality of effector cell offspring.

For immunotherapeutic approaches, it is crucial that immune effector cells are capable of entering the tumor site or malignant cell niche and are not impaired by strong immunosuppressive milieus. So far, many immunotherapies alone are not adequately effective either due to lack of sufficient numbers of effector cells, lack of sufficient activity of effector cells, or lack of MHC-I mediated antigen presentation functions. In order to overcome these struggles, combination therapies of epigenetic drugs and immunotherapies are emerging [53,54,55]. Especially, combinations of checkpoint inhibition and epigenetic modulation have demonstrated promising results. The addition of the HDACi entinostat enhanced the anti-tumor effect of an anti-PD1 therapy in murine models of lung and renal cell carcinoma. Myeloid-derived suppressor cells (MDSC) were functionally inhibited, the cytokine/chemokine release was significantly altered in vivo, and an overall transition towards a tumor-suppressive microenvironment was achieved [56]. Combining CTLA-4 blockade with the demethylating agent decitabine in a murine ovarian cancer model led to elevated differentiation of naïve into effector T cells, extended responses of cytotoxic lymphocytes, and prolonged mouse survival [57].

We hypothesized that the application of HDACi impacts the signaling between stroma and neoplastic cells, generating a more immune-permissive milieu. Here, we provide initial evidence that epigenetic modulation of stromal cells in the immediate vicinity of malignant cells may help to improve antiproliferative treatment approaches by small molecules as well as immune effector cells. So far, our studies were limited to in vitro analyses of T-MSC and a narrow source of T-MSC. Future studies should also be performed with BM-MSC from leukemia/MDS patients, and healthy donors, and consequently, must include ex vivo and in vivo analyses on effector cell functions.

## Figures and Tables

**Table 1:** Co-cultivation of PBMC with T-MSC results in an altered cytokine profile. PBMC were stimulated with IL-2/OKT3 and co-cultivated with T-MSC. Supernatants of co-cultures were analyzed after 24, 48, and 72 h. The cytokine levels were determined by Multiplex analyses. Data show mean ± SD, *n* = 4; oor <: out of range below detection level (detection levels according to standard curves for each cytokine listed in methods Section 2.3). PBMC: peripheral blood mononuclear cell; T-MSC: tumor-derived mesenchymal stromal cell; IL: interleukin; IFNγ: interferon gamma; TNFα: tumor necrosis factor alpha.

PBMC Co-Cultivation		IL-1β [pg/mL]	IL-2 [pg/mL]	IL-4 [pg/mL]	IL-10 [pg/mL]	IL-12p70 [pg/mL]	IL-15 [pg/mL]	IL-17 [pg/mL]	IFNγ [pg/mL]	TNFα [pg/mL]
**w/o T-MSC**	**24 h**	11.2 ± 6.7	1039 ± 94	3.1 ± 1.4	75.1 ± 22.7	5.3 ± 4.1	oor <	302 ± 121	74.4 ± 42.2	556 ± 272
**10,000 T-MSC**		9.1 ± 4.0	747 ± 166	2.4 ± 0.9	56.1 ± 13.9	oor <	0.52 ± 0	504 ± 209	oor <	187 ± 63
**20,000 T-MSC**		6.8 ± 5.0	859 ± 179	2.9 ± 0.6	55.2 3.7	oor <	0.63 ± 0	524 ± 189	oor <	79.2 ± 51.5
**w/o T-MSC**	**48 h**	16.6 ± 4.7	375 ± 146	4.2 ± 1.4	187 ± 29.6	6.9 ± 4.0	oor <	1269 ± 903	2187 ± 1146	2086 ± 454
**10,000 T-MSC**		31.8 ± 16.9	407 ± 141	12.9 ± 12.5	62.1 ± 18.2	7.1 ± 7.8	0.5 ± 0.08	2490 ± 1188	344 ± 255	1197 ± 991
**20,000 T-MSC**		25.3 ± 2.4	432 ± 149	11.7 ± 9.0	60.6 ± 27.7	oor <	1.4 ± 0.2	3818 ± 4133	178 ± 160	416 ± 505
**w/o T-MSC**	**72 h**	15.6 ± 2.3	179 ± 41	3.7 ± 3.1	297 ± 96.9	3.8 ± 2.8	oor <	7684 ± 5882	4754 ± 968	1294 ± 1322
**10,000 T-MSC**		59.6 ± 6.7	236 ± 58	9.6 ± 1.8	32.2 ± 15.7	1.2 ± 0	1.7 ± 0.2	3057 ± 1834	917 ± 773	457 ± 460
**20,000 T-MSC**		31.9 ± 2.9	322 ± 129	4.7 ± 1.6	23.4 ± 11.5	oor <	3.2 ± 0.6	1344 ± 949	350 ± 180	128 ± 109

**Table 2:** Cytokine production of PBMC co-cultivated with SAHA-treated T-MSC. PBMC were stimulated with IL-2/OKT3 and co-cultivated without (w/o), with control-treated T-MSC or previously SAHA-treated T-MSC. Supernatants of co-cultures were analyzed after 48 h. The cytokine levels were determined by Multiplex analyses. T-MSC of two different sources were used (T-MSC I and T-MSC II). PBMC of two different healthy donors were co-cultivated with each T-MSC in two separate experiments. Data show mean ± SD, *n* = 4. PBMC: peripheral blood mononuclear cell; T-MSC: tumor-derived mesenchymal stromal cell; IL: interleukin; IFNγ: interferon gamma; TNFα: tumor necrosis factor alpha; SAHA: suberoylanilide hydroxamic acid.

PBMC Co-Cultivation	Treatment of T-MSC	IL-1β [pg/mL]		IL-2 [pg/mL]		IL-10 [pg/mL]		IL-17 [pg/mL]		IFNγ [pg/mL]		TNFα [pg/mL]	
		**T-MSC I**	**T-MSC II**	**T-MSC I**	**T-MSC II**	**T-MSC I**	**T-MSC II**	**T-MSC I**	**T-MSC II**	**T-MSC I**	**T-MSC II**	**T-MSC I**	**T-MSC II**
**w/o T-MSC**		14.3 ± 3.4		1135 ± 222		232 ± 26.3		79.1 ± 12.7		741 ± 446		2178 ± 453	
**20,000** **T-MSC**	**0 µM SAHA**	686 ± 382	335 ± 282	839 ± 398	978 ± 85.4	288 ± 125	198 ± 64.0	185 ± 64.3	140 ± 52.9	869 ± 690	124 ± 124	120 ± 102	15.2 ± 14.0
	**0.1 µM SAHA**	496 ± 81.4	411 ± 409	801 ± 303	987 ± 162	281 ± 85.8	170 ± 51	161 ± 118	260 ± 168	875 ± 436	323 ± 201	77.4 ± 25.3	20.0 ± 12.7
	**0.2 µM SAHA**	736 ± 111	506 ± 524	816 ± 270	844 ± 118	305 ± 142	186 ± 102	118 ± 71.9	198 ± 133	1090 ± 306	427 ± 284	125 ± 10.5	15.9 ± 1.1

## List of Abbreviations

AML	Acute myeloid leukemia
BM-MSC	Bone marrow-derived mesenchymal stromal cell
CAR	Chimeric antigen receptor
CLL	Chronic lymphocytic leukemia
FDA	United States Food and Drug Administration
HDACi	Histone deacetylase inhibitors
HPC	Hematopoietic progenitor cells
IFNγ	Interferon-gamma
IL	Interleukin
MDS	Myelodysplastic syndrome
MDSC	Myeloid-derived suppressor cell
MFI	Mean fluorescence intensity
MHC	Major histocompatibility complex
MSC	Mesenchymal stromal cells
NOS	Nitric oxide synthase
PBMC	Peripheral blood mononuclear cell
SAHA	Suberoylanilide hydroxamic acid
T-MSC	Tumor-derived mesenchymal stromal cell
TNFα	Tumor necrosis factor alpha
